# A Two-Phase Cross-Modality Fusion Network for Robust 3D Object Detection

**DOI:** 10.3390/s20216043

**Published:** 2020-10-23

**Authors:** Yujun Jiao, Zhishuai Yin

**Affiliations:** 1 School of Automotive Engineering, Wuhan University of Technology, Wuhan 430070, China; jacky723@whut.edu.cn; 2 Hubei Key Laboratory of Advanced Technology for Automotive Components, Wuhan University of Technology, Wuhan 430070, China; 3 Hubei Research Center for New Energy & Intelligent Connected Vehicle, Wuhan 430070, China

**Keywords:** 3D object detection, cross-modality fusion, deep convolutional neural networks

## Abstract

A two-phase cross-modality fusion detector is proposed in this study for robust and high-precision 3D object detection with RGB images and LiDAR point clouds. First, a two-stream fusion network is built into the framework of Faster RCNN to perform accurate and robust 2D detection. The visible stream takes the RGB images as inputs, while the intensity stream is fed with the intensity maps which are generated by projecting the reflection intensity of point clouds to the front view. A multi-layer feature-level fusion scheme is designed to merge multi-modal features across multiple layers in order to enhance the expressiveness and robustness of the produced features upon which region proposals are generated. Second, a decision-level fusion is implemented by projecting 2D proposals to the space of the point cloud to generate 3D frustums, on the basis of which the second-phase 3D detector is built to accomplish instance segmentation and 3D-box regression on the filtered point cloud. The results on the KITTI benchmark show that features extracted from RGB images and intensity maps complement each other, and our proposed detector achieves state-of-the-art performance on 3D object detection with a substantially lower running time as compared to available competitors.

## 1. Introduction

As a crucial task in various engineering applications including autonomous driving, safety management, et cetera, high-precision object detection has drawn a great deal of attention in recent years. Two sources of inputs are commonly used in objection detection: RGB images and LiDAR point clouds.

A large number of deep learning-based models such as the series of Faster RCNN, SSD, YOLO [1,2,3], and a lot more custom versions of them have been developed for 2D object detection with RGB images. Despite tremendous progresses made in the past few years, vision-based 2D detectors still have major limitations, especially when they are developed for applications such as autonomous driving, where failures of the detector can have disastrous consequences [4]. The vulnerability to environmental interference as well as the lack of depth information are major drawbacks and inherent deficiencies of vision-based 2D detectors, which can hardly be remedied without employing different modalities of data [5,6,7].

In terms of 3D object detection with point clouds, a breakthrough is made with the introduction of Pointnet [8] which enables direct 3D objection on disordered raw point clouds without prior knowledge. Though, the sparseness of the point cloud and the high computational overhead impacts both the detection accuracy and the real-time performance of point cloud-based 3D detection.

Cross-modality fusion which combines aforementioned two modalities of information for the purpose of more precise and robust object detection has therefore become a research focus.

Most schemes of cross-modality fusion are implemented at three different stages or levels. The raw data-level fusion [9,10], which is an early-stage fusion, superimposes data from multiple sensors, producing a significantly larger amount of input data upon which the extracted features are not guaranteed to be more expressive. The decision-level fusion [11,12,13], which is a late-stage fusion, is hindered by critical issues such as difficulties in obtaining prior knowledge, loss of information, et cetera. The intermediate feature-level fusion [11,14], which merges the features extracted from each modality of data to produce more robust and informative fused features, is intuitively considered as a more effective way of exploiting useful multi-modal information. However, fusing multi-modal features remains a non-trivial task mainly due to two reasons: (1) the features extracted from the image and the point cloud differ greatly in many perspectives including the point of view, the density, the level of semantics and spatial details, et cetera. As a result, it is challenging to build a good correspondence between multi-modal features; (2) each modal of inputs is vulnerable to changes in different influencing factors in the surrounding environment. It is quite difficult, yet essential, to design a fusion strategy that is capable of combining meaningful multi-modal features while eliminating mutual interference under ever-changing environmental conditions.

Therefore, we propose to develop an effective and efficient fusion scheme that implements cross-modality fusion at both the decision-level and the feature-level to avoid information loss and having to devise a complicated yet possibly ineffective fusion strategy. For decision-level fusion, inspired by the Frustum Pointnet [12], which brings forward a method that uses images to assist point cloud based object detection, we propose a novel two-phase cascaded detector with the first-phase being a 2D detector that produces proposals. The 2D proposals are used to filter LiDAR point clouds, so that only the points that reside within the regions of interest are fed into the second-phase 3D detector. Since the performance of the two-phase cascaded detector is bounded by each stage, it is essential to further enhance the detection performance of the first-phase 2D detector. Furthermore, with only RGB images used for 2D detection in the first phase, the complementarity among different sensors are not fully exploited. Therefore, we propose implementing a feature-level fusion in the first-phase and building the 2D detector in the framework of a two-stream Faster R-CNN [1] to extract and fuse features from RGB images and intensity maps, which are generated by projecting the reflection intensity values of LiDAR point clouds to the front view plane. The aim of this design is to produce more robust and expressive features upon which more accurate object classification and bounding box regression can be achieved. The intensity map is chosen as the complementary source of input, because as pointed out in MCF3D [9], the reflection intensity value represents the materials and depths of objects in a certain extent, and is immune to changes in weather and lighting conditions. However, instead of concatenating RGB images and intensity maps to produce fused raw data as is done in MCF3D [9], we believe that a feature-level fusion is more effective in preserving useful information while eliminating cross-modality interference. A cascade detector head is then implemented for classification and bounding box regression, aiming to improve the detection performance on small targets. Finally, the 2D proposals generated by the first-phase detector are transformed into the point cloud space so as to acquire the corresponding regions of interest in the point cloud. The filtered point cloud is classified and segmented with the Point-Voxel Convolution Network (PVConvNet) [15] to produce 3D detection results. Experimental results on the KITTI benchmark dataset [16] show that our proposed detector achieves state-of-the-art performance when compared to available competitors.

Main contributions of our work are summarized as follows:

1. We propose a cascading 3D detector that exploits multi-modal information at both the feature fusion and decision making levels.
2. At the decision-level, we design a two-phase detector in which the second-phase 3D detection gets assistance from the first-phase 2D detection in a way that 2D detection results are transformed into 3D frustums to filter the point cloud, in order to improve both the detection accuracy and real-time performance of 3D detection.
3. At the feature-level, we design a two-stream fusion network to merge cross-modality features extracted from RGB images and intensity maps, so as to produce more expressive and robust features for high-precision 2D detection. The validity of the proposed feature fusion scheme is examined and strongly supported by the experimental results and through visualizing features at multiple network stages.

## 2. Related Work

### 2.1. 2D Object Detection with Images

Objection detection has been a fundamental task in the field of computer vision for decades.

Motivated by the success of Alexnet [17] on image classification, a great number of 2D detectors based on deep CNNs have been developed in the past few years. Depending on whether region proposals are involved, most detectors can be classified into two categories: (1) two-stage detectors which are proposal-based and anchor-based, including Faster RCNN, R-FCN, et cetera. [1,18] that use a region proposal network (RPN) to generate proposals for detection. (2) one-stage detectors which are proposal-free, including YOLO, SSD, et cetera. [2,3], that regard object detection as a regression problem which is solved with end-to-end convolution networks.

To improve the performance of vision-based 2D detectors, extensive efforts have been made from different perspectives. As the most representative work in exploring the effectiveness of multi-layer fusion, Feature pyramid networks (FPN) [19] proposes producing more robust and expressive features that comprise high-level semantics and low-level spatial details by introducing the top-down structure with lateral connections from the bottom-up feature extraction backbone. R-FCN [18] introduces the position-sensitive score map to resolve the conflict between translation invariance in classification and translation variance in object detection, for more accurate and faster detection; Cascade-RCNN [20] implements multi-stage detection with progressively higher thresholds for the balance of better performance and high Intersection over union (IoU).

### 2.2. 3D Object Detection with Point Clouds

Depending on how the point cloud is represented, studies on 3D object detection fall into three categories: (1) bird’s-eye view (BEV) and front-view (FV) based methods [21,22,23,24,25]. MV3D [23] is the first study to project the point cloud onto the ground plane to create the BEV. Li et al. [25] converts the point clouds into front-view 2D maps. On the basis of projected 2D maps, 2D-CNN is applied to generate region proposals as done in vision-based 2D detectors. (2) Voxel based methods [26,27,28,29,30], which transforms the point clouds into voxels by dividing the point clouds into three-dimensional voxel grids with spatial dependencies. Three-dimensional CNNs are then applied to generate region proposals. (3) Raw point cloud-based methods. The milestone works are Pointnet [8] and its improved version Pointnet++ [31]. These methods operate directly on the point clouds to extract local features layer by layer at different scales and obtain deep features through a multilayer network. Afterwards, a number of variants of Pointnet [8] are proposed to further improve the performance of point cloud-based 3D detection from different perspectives. A-CNN [32] proposes an annular convolution to capture the local neighborhood geometry of each point that adapts to the variability and scalability of point cloud, improving the performance of the point-based model. GAPnet [33] learns local geometric representations of point-wise and its neighborhood by implementing graph attention mechanisms to enhance network robustness. RS-Pointnet [34] learns features and shapes from geometric topology constraint among points to obtain contextual shape-aware information for object detection and segmentation.

It is worth mentioning that a number of knowledge-based methods [35,36] have also been proposed to operate directly on raw point clouds. Traditionally, since 3D point cloud learning is conducted based on scenario-specific knowledge-base and ontologies built artificially, the performance and generalization ability of such approaches when applied in object detection in scenes that are highly dynamic and diverse is not satisfying. Though, with the mechanism of self-learning introduced, recent approaches [37,38] have shown promising improvements since ontologies are updated continuously and automatically.

As pointed out in previous studies [15,39], voxel-based and point-based representation each have their benefits and drawbacks. Voxel-based methods generate high-quality proposals but suffer from information loss. A high-resolution representation is required in order to preserve fine details for high-quality feature extraction. However, the computational overhead and memory usage increase cubically with the voxel resolution. Point-based methods are flexible in receptive fields but structuring the sparse point cloud is inefficient. PV-RCNN [15] proposes the PVConv that disentangles the voxel-based branch and the point-based branch, in which the former branch utilizes voxel-CNN to aggregate the neighboring points in low-resolution voxel grids and the latter one extracts the features from each point with high resolution. PV-RCNN [15] first uses the 3D voxel-CNN with multi-scale layers to generate proposals, while the voxel set abstraction module extracts the features of key-points by FPS (farthest point sampling) for points in voxels. Approaches proposed in the two aforementioned researches attempted to improve the efficiency and precision of 3D object detection by integrating the advantages of voxel-based and point-based methods, which inspires the design of our second phase 3D detector.

### 2.3. Object Detection Based on Multi-Modal Fusion

To compensate for the limitations of a single or a single-modal sensor in complex and dynamic conditions, multi-sensor fusion has become a research focus in the field of objection detection. There are two main research foci: fusion of cross-spectral images for 2D detection, and fusion of visible images and point clouds for 3D detection.

For robust all-day 2D detection, numerous studies have proposed fusing cross-spectral images mainly from the visible and infrared spectrums [40,41,42,43]. Although the modal of inputs is different from those of our work, mechanisms such as attention based adaptive weighting, the framework of multiple streams of feature extraction, et cetera, are all quotable approaches in designing multi-modal fusion networks.

For 3D detection, although some studies such as Stereo-RCNN [44] focused on exploring the fusion structure to process stereo images for 3D detection, most researches focus on fusing visible images and point clouds [9,12,14,22,23,45].

Similar to studies done in the field of point cloud-based 3D detection, in order to establish a corresponding relation between multi-modal inputs so that a fusion operation is applicable, voxelization and projection of LiDAR point cloud is often considered as an indispensable step in the design of many previously proposed fusion methods. MV3D [23] implements the fusion by projecting 3D object proposals generated from the BEV feature maps to the feature maps extracted from the front view and RGB images respectively. Region-wise features obtained are then fused via ROI pooling for final detection. AVOD [22] improves MV3D [23] by eliminating the LiDAR FV branch and introducing a decision-level fusion on top of the existing feature-level fusion. Wang et al. [46] fuses the BEV LiDAR data and the front view image at the feature-level with non-homogeneous pooling, followed by a one-stage RPN.

Attempts have also been made to implement the multi-modal fusion in the intermediate feature level by projecting image semantics into the space of the point cloud to enhance the point features. Liang et al. [21] proposes projecting the multi-scale intermediate image features into BEV with continuous convolutions to generate dense BEV feature maps upon which 3D detection is performed. Three-dimensional-CVF [5] designs a cross-view feature fusion strategy which transforms the 2D image features to a smooth spatial feature map corresponding to the LiDAR features in the BEV view. EPNet [6] introduces a novel grid generator to establish point-wise correspondence between LiDAR and image data without having to generate BEV data.

To implement an adaptively weighted fusion of multi-modal information in the intermediate feature levels so that the detector is robust to environmental changes, weighting mechanisms that determines the contribution of features from each modality are studied. Kim et al. [47] employs the gated information fusion network to adjust the weights of the features from each modality so that degraded features are suppressed. 3D-CVF [5] develops a gated feature fusion network based on spatial-wise attention to generate the fused feature. EPNet [6] adaptively estimates the importance of image features utilizing the LiDAR feature and weights the semantic image features before they are employed to enhance the corresponding LiDAR point features at multiple network layers.

Multi-modal fusion schemes that are built upon PointNet [8,31] which operate directly on point cloud have also been proposed. PointFusion [14] fuses the image’s features, point-wise and global features of the point cloud, and utilizes two different fusion networks to predict object classes and regress bounding boxes. F-Pointnet [12] implements the multi-modal fusion in a cascaded manner in which different modalities of inputs are used in different phases.

## 3. Methods

### 3.1. Overview

**Figure 1:**
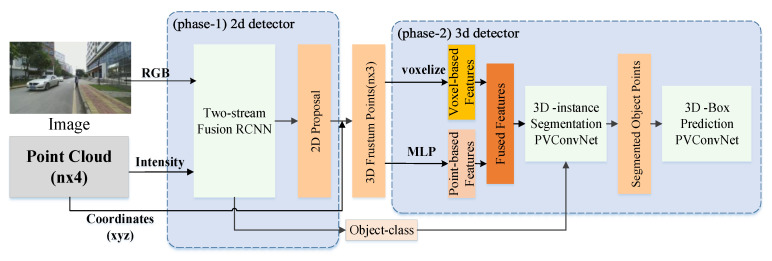
The diagram of the multi-phase fusion network.

We propose a novel two-phase 3D object detector in which cross-modality fusion of RGB images and point clouds are implemented at both the feature-level and the decision-level. As illustrated in Figure 1, our two-phase 3D detector comprises of phase-1: a two-stream fusion RCNN which merges RGB images and intensity maps at the feature-level and produces 2D proposals to generate 3D frustums in the space of the point cloud, and phase-2: a PVConvNet-based 3D detector which performs 3D instance segmentation and box regression on point clouds in the 3D frustums.

### 3.2. Two-Stream Fusion RCNN

Instead of concatenating the RGB image and the intensity map to obtain an RGB-I representation of the scene, we argue that a feature-level fusion would contribute to merging more expressive and useful information and therefore producing more informative and robust representations of the perceived scene.

**Figure 2:**
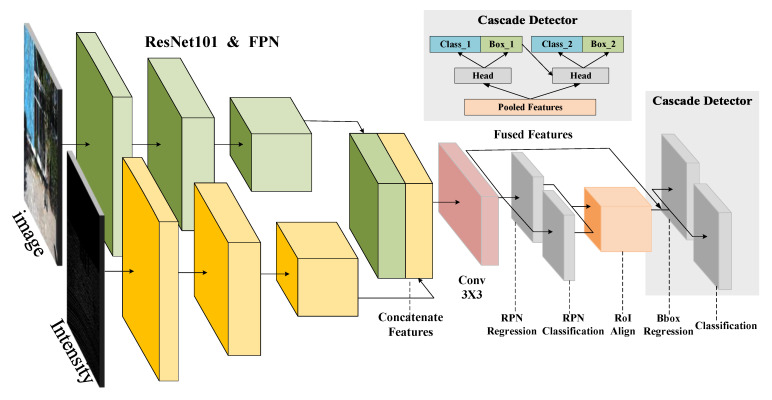
The two-stream fusion RCNN.

As shown in Figure 2, we built two streams of feature extraction with ResNet101 [48] to extract features from the RGB images and the intensity maps, respectively.

Features extracted at the same stage are concatenated to produce fused features. Moreover, the fusion process is implemented based on the FPN structure [19] so that multiple stages of fused features are combined in order to preserve both low-level details and high-level semantics.

Specifically, the extracted multi-scale features are input into the modified RPN to generate proposals. The modified RPN network consists of a 3 × 3 convolution layer followed by ReLU activation and two sibling fully-connected layers to classify objects and regress anchor boxes. Proposals are generated by sliding anchors on multiple scales of features and are then concatenated as the outputs. Together with proposals, the fused multi-scale features are fed into the PyramidRoI pooling layer to fuse the semantic information at different levels and scales. The fused multi-scale semantics are then fed into the top model of the first cascaded head to predict the class of objects and regress their bounding boxes.

Inspired by Cascade RCNN [20], we design a cascade detector head to further improve the detection performance, especially on small targets. Each detector head is comprised of two convolutional layers followed by ReLu activation and two fully-connected layers for classification and regression. In the sequence of detector heads, the predicted boxes of the previous stage are filtered with Non maximum suppression (NMS) and are then served as proposals of the following stage. The threshold of IoU is increased along with the depth of the detector head so that the network tends to focus more on small-scale targets.

Besides, we explore the effect of weighted fusion based on attention and present the results in Section 4.3. The attention module is implemented based on CBAM [49], which consists of a channel-wise and a spatial-wise attention module, and is incorporated into the backbone network of each stream. The channel-wise attention module employs the global max-pooling and global average pooling to process each scale of feature maps, which are then fed into a shared Multi-layer Perceptron (MLP) followed by a sigmoid module to generate channel-wise attention values. The spatial-wise attention module employs the same pooling operation as in the channel-wise attention module to process the feature maps, upon which convolutional operations and sigmoid activations are applied to produce spatial-wise attention values.

### 3.3. PVConvNet-Based Object Detection

With the assistance of 2D detection, we implement a PVConvNet-based 3D detector to process the point clouds of interest in frustums, which are basically 3D transformations of 2D bounding boxes. PV-CNN [15] combines the advantages of Pointnet [8,31] and voxel models [26,27], and improves the accuracy of positioning the object in the point cloud and identifying the scene more efficiently. We adopt the PVConvNet to complete detection task on filtered point clouds, including point-voxel convolution, 3D instance segmentation and 3D box estimation.

#### 3.3.1. Point-Voxel Convolution

The point-voxel convolution contains two branches as shown in Figure 1. One is the voxel-based branch with good data locality and regularity, and the other is the point-based branch. The voxel-based branch transforms the points into low-resolution voxel grids and aggregates the neighboring points with voxel-based convolutions, and then it converts voxels back to points by devoxelization. The point-based branch extracts the features for each individual point.

#### 3.3.2. 3D Detection

With the fused features obtained from voxel-based and point-based branches, we implement the 3D instance segmentation and 3D box estimation as did in F-Pointnet [12] to produce the final output. Similar to 2D instance segmentation which is a binary classification of each pixel, 3D instance segmentation classifies point clouds and predicts the confidence that a point is part of an object of interest. In our implementation, we encode the object category from the two-stream fusion RCNN into one-hot class features vector, and concatenate them with the point cloud features learned by the 3D detection model. Having obtained the segmentation results, we convert the point cloud to the local coordinate system and utilize PointNet [8] to perform more accurate box estimation.

## 4. Experiments

### 4.1. Experimental Setups

The KITTI vision benchmark suite [16] is used to evaluate our proposal. As done in F-Pointnet [12], we divided a total of 7481 images and corresponding point clouds into two subsets with roughly the same size, as the training and the testing dataset, respectively. All objects were subcategorized into ”easy”, ”moderate” and ”hard” according to the heights of the 2D bounding boxes, levels of occlusion and truncation. The intensity values of the point cloud were extracted, transformed and projected onto the front view plane in the coordinate system of the camera to generate intensity maps. The kitti-object-eval-python script is used to calculate the AP (average precision), which is used as the metrics to measure the detection performance of our work and comparable detectors.

### 4.2. Implementation Details

For the two-stream fusion RCNN, the pre-trained RestNet101 [48] model on ImageNet [50] was used to initialize the backbone network of feature extraction. It was then trained on the KITTI training set using SGD [51] with a weight decay of 0.0005, a momentum of 0.9 and a batch size of 1 on a 4 Titan XP GPU server (Nvidia, Santa Clara, CA, U.S.A.). The learning rate was set to 1 × 10^−3^ in the first 10 epochs and was decreased to 1 × 10^−4^ in the last 4 epochs. Other implementation details were the same as the original Faster RCNN [1]. For the PVConvNet, we used the training data prepared in F-Pointnet [12] to train the network, which contained over 7000 pairs of color images and frames of filtered point cloud data. The 3D detector was trained using the Adam optimizer [52] with a learning rate of 1 × 10^−3^ on a 4 Titan XP GPU server with batch size of 32 and epochs of 200.

### 4.3. Ablation Study

#### 4.3.1. Cross-Modality Fusion

To verify the effectiveness of feature-level fusion of RGB images and intensity maps in enhancing the expressiveness of the merged features, we compared the performance of our two-stream fusion RCNN (VI-fusion) and the baseline Faster RCNN [1]. The results presented in Table 1 show significant improvements in detection performance in all categories of objects at all levels of difficulties.

**Figure 3:**
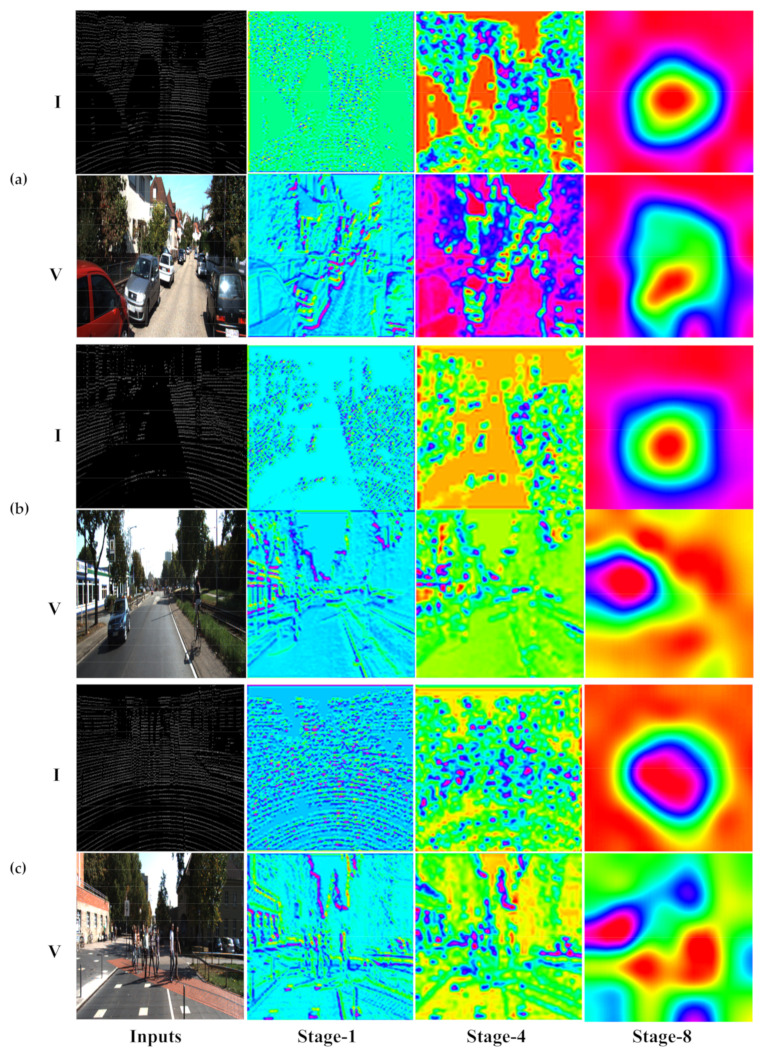
Visualized feature maps at multiple stages from two modalities. (**a**), (**b**) and (**c**) each present a test scene. In each subfigure, the V-labeled row present the input and the feature maps of the RGB stream, while the I-labeled row present those of the intensity stream.

In Figure 3, we visualize the feature maps generated at stage 1, stage 4 and stage 8 in both network streams for more discoveries as to why merging features from RGB images and intensity maps contribute to producing more informative and robust features.

The V-labeled rows present feature maps of the RGB stream, while the I-labeled rows present those of the intensity stream.

Observations from the visualized feature maps are three-fold: First, as shown in Figure 3a,b, while RGB features at stage 4 seem to have lost most visual details, intensity features at stage 4 outlines objects rather clearly, meaning fine visual details are still preserved in the intensity stream. As a result, merging RGB and intensity features at the same stage is beneficial since they not only represent entirely different sets of physical information, but also contain different levels of semantics and visual details of the same scene. Second, as shown in the 4th column of Figure 3b, the area of the car is attended to in the RGB feature map, while the area of the cyclist is considered as less relevant to the task of detection. In contrast, a better overall representation of all objects is obtained in the intensity feature map since the region of interest encompasses both objects without having the most conspicuous object being overwhelmingly dominant. Third, although the intensity feature map preserves much less visual detail of smaller targets, such as pedestrians and cyclists, due to the sparsity of point clouds, it is clearly shown in Figure 3c that the intensity feature provides a more proper description of the area that the detector should attend to, as opposed to biased attention caused by RGB features.

It was also discovered in our experiments that the intensity of the point cloud is subject to changes in the materials and the micro-structures of the objects. Features extracted from the intensity maps are therefore less robust when the objects of interest are highly diverse in terms of these two factors.

#### 4.3.2. Cascade Detector Head and Attention-Based Weighted Fusion

To examine the effectiveness of the cascade network head, we increased the stage of heads gradually from 1 to 3 and evaluated the performance of 3D detection. As shown in Table 2, Model-v2, which was equipped with 2 heads outperformed Model-v1 and Model-v3, which were equipped with 1 and 2 network heads, respectively.

Model-v1-att indicates that the attention module was implemented with 1 detector head. Model-v2-att indicates that the attention module was implemented with 2 detector heads. It was observed that having a second stage of network heads helps to significantly improve the overall performance of the detector, especially on small-scale targets. However, a deeper cascade structure does not lead to further improvement in detection performance. As a result, we adopted the two-stage cascade design in our 2D detector.

As for the detectors with attention modules attached, a performance degradation instead of an improvement was seen. It was proven by the results that although weighted fusion is intuitively believed to be beneficial, the design of the weighting mechanism is challenging. Attention mechanisms are not necessarily contributive in enhancing the fused feature since it is a non-trivial task to devise a strategy that is capable of adaptively adjusting the contribution of multi-modal features whose qualities are subject to changes in numerous complex environmental factors.

### 4.4. Comparison with Other Methods

The detection performance of our proposal and 9 available competitors are compared and the results are given in Table 3 and Table 4.

**Figure 4:**
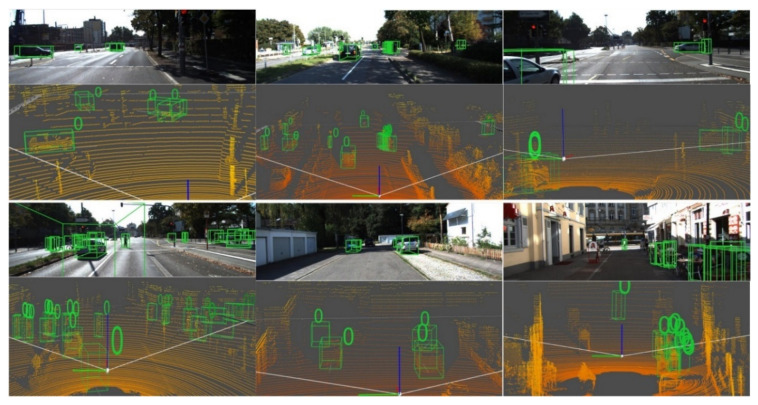
Visualization of 3D Detection Results.

Figure 4 shows some visualized results of 3D detection.

As shown in Table 3 and Table 4, our work is proven to be a state-of-the-art detector as it achieves the best detection performance in 1 subcategory and approaches the best in the other 8, at the cost of the least computation time. The Range RCNN [56], Deformable RCNN [57], and STD [58] each lead in the detection of 1 or 2 subcategories of cars and cyclists; however, they perform much worse in all subcategories of pedestrians as compared to ours. Our proposal outperforms F-PointNet++ [12] in detection of all subcategories of cars and ”easy” pedestrians, while rivaling its performance in all other subcategories of objects.

The results suggest that the 3D detection methods, which uses point cloud as the only input, perform well in the detection of objects such as cars and cyclists, which have regular structures and robust geometric features, while performing poorly in detecting pedestrians with far more diverse appearances and geometries, due to the absence of abundant texture and semantic features from images.

## 5. Conclusions

For robust and efficient 3D object detection, we propose a novel two-phase fusion network which exploits cross-modality information from RGB images and LiDAR point clouds at both the feature-level and the decision-level. The results of comparison between our proposal and the baseline Faster RCNN strongly support the assumption that a cross-modality fusion at the feature-level contributes effectively towards enhancing the expressiveness and robustness of the fused features and consequently improving the performance of detection on all subcategories of objects. We investigated the underlying causes by visualizing feature maps at multiple stages from both modalities. It was discovered that the intensity feature still preserves fine visual details which are hardly observable in the corresponding RGB feature at the same network stage. Moreover, it is shown that at least in some cases, intensity features help to refine or adjust the area that the network attends to and therefore a more proper overall representation of all objects of interest is obtainable. Compared to available state-of-the-art competitors, our proposal achieves either the best or near the best detection accuracy in multiple categories of objects while significantly outperforming real-time performance. Future studies will investigate more robust 2D representation of point clouds to further improve the performance of first-phase 2D detection.

## Figures and Tables

**Table 1:** Comparison of the baseline Faster RCNN and the two-stream fusion RCNN average precision (AP).

Models.	Car			Pedestrian			Cyclists		
	Easy	Moderate	Hard	Easy	Moderate	Hard	Easy	Moderate	Hard
Baseline	92.12	87.10	75.61	79.20	69.85	60.59	78.95	61.50	56.53
VI-fusion	97.99	90.70	82.43	83.26	76.42	65.84	82.07	70.50	61.53

**Table 2:** Comparison of different depth of cascaded detector heads (AP).

Models	Car			Pedestrian			Cyclists		
	Easy	Moderate	Hard	Easy	Moderate	Hard	Easy	Moderate	Hard
Model-v1	97.99	90.70	82.43	83.26	76.42	65.84	82.07	70.50	61.53
Model-v2	**98.83**	**95.18**	**84.86**	**86.30**	**78.28**	**70.65**	**84.20**	**71.33**	**63.25**
Model-v3	97.61	92.42	81.36	84.12	76.47	67.51	83.33	69.38	60.41
Model-v1-att	94.79	88.36	80.62	82.86	73.98	62.86	78.71	58.30	56.83
Model-v2-att	95.11	90.12	82.05	85.40	74.45	64.65	81.60	59.83	57.05

The top performance networks are marked in bold

**Table 3:** The mean average precision (mAP) of different models on the KITTI dataset (AP).

Method.	Cars			Pedestrians			Cyclists		
	Easy	Moderate	Hard	Easy	Moderate	Hard	Easy	Moderate	Hard
MMLab–PointRCNN [53]	83.68	72.31	63.17	46.68	39.34	36.01	74.83	58.65	52.37
MV3D [23]	74.97	63.63	54.00	-	-	-	-	-	-
AVOD [22]	76.39	66.47	60.23	36.10	27.86	25.76	57.19	42.08	38.29
PointGNN [54]	88.33	79.47	72.29	51.92	43.77	40.14	78.60	63.48	57.08
Frustum ConvNet [55]	87.36	76.39	66.9	52.16	43.38	38.80	78.68	65.07	56.54
F-Pointnet++ [12]	82.15	68.47	62.07	64.02	**53.84**	**49.17**	72.27	56.12	50.06
Range RCNN [56]	**88.47**	81.33	**77.09**	-	-	-	-	-	-
Deformable PV-RCNN [57]	83.30	**81.46**	76.96	46.97	40.89	38.80	73.46	**68.54**	**61.33**
STD [58]	86.61	77.63	76.06	53.08	44.24	41.97	**78.89**	62.53	55.77
Ours (v2)	84.05	71.50	63.01	**64.26**	52.71	48.72	72.15	55.70	49.24

The top performance networks are marked in bold

**Table 4:** The real-time performance of different models on the KITTI dataset.

Method	Input Data	Latency
MMLab–PointRCNN [53]	Point	112.4 ms/frame
MV3D [23]	Point + RGB	360 ms/frame
AVOD [22]	Point + RGB	80 ms/frame
PointGNN [54]	Point	80 ms/frame
Frustum ConvNet [55]	Point + RGB	470 ms/frame
F-Pointnet++ [12]	Point + RGB	97.3 ms/frame
Range RCNN [56]	Range	60 ms/frame
PV-RCNN [57]	Point	80 ms/frame
STD [58]	Point	80 ms/frame
Ours (v2)	Point + RGB + Intensity	59.6 ms/frame
